# The effect of microbial activity on soil water diffusivity

**DOI:** 10.1111/ejss.12535

**Published:** 2018-01-30

**Authors:** B. U. Choudhury, S. Ferraris, R. W. Ashton, D. S. Powlson, W. R. Whalley

**Affiliations:** ^1^ Department of Sustainable Agricultural Sciences Rothamsted Research Harpenden AL5 2JQ UK; ^2^ Division of Natural Resource Management ICAR (RC) for NEH Region, Umiam, Ri‐Bhoi Meghalaya 793 103 India; ^3^ Politecnico and University of Torino Interuniversity Department of Regional and Urban Studies and Planning, Viale Mattioli 39 Torino Italy

## Abstract

In this study, we explored the effects of microbial activity on the evaporation of water from cores of a sandy soil under laboratory conditions. We applied treatments to stimulate microbial activity by adding different amounts of synthetic analogue root exudates. For comparison, we used soil samples without synthetic root exudates as control and samples treated with mercuric chloride to suppress microbial activity. Our results suggest that increasing microbial activity reduces the rate of evaporation from soil. Estimated diffusivities in soil with the largest amounts of added root exudates were one third of those estimated in samples where microbial activity was suppressed by adding mercuric chloride. We discuss the effect of our results with respect to water uptake by roots.

**Highlights:**

- We explored effects of microbial activity on the evaporation of water from cores of a sandy soil.
- We found the effect of microbial activity on water release characteristic was small.
- Increasing microbial activity reduced evaporation from soil, while microbial suppression increased it.
- Effect of microbial activity on root water uptake was estimated to be equivalent to a change in soil structure.

## Introduction

Microbial activity can greatly affect the structure and hydraulic properties of soil (e.g. Or *et al*., 2007; Colica *et al*., 2014; Helliwell *et al*., 2014). One of the explanations for these effects is related to the production of extracellular polymeric substances that alter soil structure at the pore scale (Or *et al*., 2007). Typically, a smaller hydraulic conductivity is reported and explained by the clogging of pores because of microbial activity. An important stimulus of microbial activity in soil is the carbon released from roots in the form of root exudates (Paterson *et al*., 2007). Root exudates can have immediate effects on the soil water release characteristics because of their surfactant properties (Read *et al*., 2003). In the longer term, incubation of soil with root exudates, either natural or synthetic, has been shown to reduce hydraulic conductivity in near‐saturated soil (Hallett *et al*., 2003; Whalley *et al*., 2004). The study of Colica *et al*. (2014) showed that induced biological crusts could reduce the rate of evaporation from dry soil. They also found that the hydraulic conductivity of near‐saturated soil depended on the molecular weight of carbohydrates added to soil; the hydraulic conductivity was less when higher‐molecular‐weight carbohydrates were added.

The purpose of this study was to determine the effect of root exudates on soil water release curves and hydraulic conductivity over a wide range of soil water contents. We used a sandy soil, as did Colica *et al*. (2014), to minimize the effects of complex changes in soil structure that can be induced by microbial activity. The hydraulic properties of relatively dry soil were determined by measuring the rate of evaporation and the data were interpreted with a simple analytical solution to evaporation from bare soil (Black *et al*., 1969; Parlange *et al*., 1992). The effects of stimulating microbial activity on soil hydraulic properties have been reported previously, whereas the effects of suppressing microbial activity have received less attention. In our study, we included soil treated with added mercuric chloride to suppress microbial activity. We tested the hypothesis that soil with an active microbial population was less conductive to water. We used our data to investigate the likely effect of microbial activity on water uptake by roots.

## Materials and methods

### *Soil sample preparation*

Soil samples from the surface layer (0 to 20 cm) were collected during April 2015 from three ploughed fallow plots of a randomized experiment on Butt Close experimental field, Woburn Experimental Farm (52° 00′ 42″ N, 0° 32′ 42″ W), Rothamsted Research, UK. Each of the plots was in a separate block as described by Shanahan *et al*. (2015). Butt Close soil is a loamy sand soil (sand: 87.5%, silt: 5.5% and clay: 7.2%), and taxonomically (FAO) this soil is classified as an Arenosol. It has a small organic carbon content (1%), near neutral soil pH (6.63, 1:2 soil:water ratio) and a particle density of 2.65 g cm^−3^ (Whalley *et al*., 2008).

Three soil samples from each plot were air‐dried, ground and sieved through a 2‐mm sieve separately and treated as replicates in the laboratory studies. The moisture content of air‐dried soil samples was 1% (weight by weight). Soil was packed into stainless cores (3.59 cm long and 3.86 cm in diameter). The bases of the cores were covered with a fine nylon cloth. The cores were filled homogenously with air‐dried 2‐mm‐sieved loamy sand soil to a bulk density of 1.5 g cm^−3^.

We prepared artificial root exudates of low‐molecular‐weight organic compounds from the mixtures of 15 compounds (Paterson *et al*., 2007) comprising five carbohydrates (glucose, fructose, sucrose, arabinose and ribose), five amino acids (glycine, valine, glutamine, serine and alanine) and five organic acids (malic, citric, malonic, oxalic and fumaric). A stock solution of 4.166% C concentration (41.66 g l^−1^) was prepared by dissolving each of these 15 compounds (equal in terms of C content: 1.39 g) in 500 ml of distilled water. From this stock solution, three different working solutions of root exudates were prepared with enough distilled water to maintain the soil at 100% of water‐holding capacity while ensuring an enrichment of 1.25, 2.5 and 5.0 g C kg^−1^ in dry soil packed at a density of 1.5 g cm^−3^. Stainless steel cores were filled with air‐dried sieved soil in triplicate and they were saturated with the three solutions for 48 hours at 20°C together with a control (without root exudates). The control was saturated in distilled water. Saturation was achieved by placing the samples on a Haynes apparatus and slowly raising the water table to obtain uniform saturation across each core.

To stop microbial activity, we applied two additional treatments: (i) soil amended with mercuric chloride solution in one root exudates mixture (2.5 g C kg^−1^ dry soil) and (ii) the soil with distilled water was also amended with mercuric chloride solution. Among the several commonly used soil sterilizing methods in laboratory experiments, mercuric chloride results in effective sterilization with minimal effect on soil chemical and physical properties (Wolf *et al*., 1989; Wang *et al*., 2011), and more importantly the sterile environment lasts for at least 3 weeks (Tuominen *et al*., 1994; Stephens *et al*., 2002). Although formaldehyde is more effective at preventing microbial activity, the effectiveness of mercuric chloride is comparable (Tuominen *et al*., 1994). Importantly for this study, however, mercuric chloride is not a solvent that will evaporate and confound our soil evaporation data (see below). Two sterilized treatments with mercuric chloride solution of 0.1% were prepared by dissolving 0.78 g HgCl_2_ in 250 cm^−3^ of the previously prepared working solution of root exudates (2.5 g C kg^−1^ dry soil) and in distilled water. Soil, also packed to a bulk density of 1.5 g cm^−3^, was saturated in 0.1% mercuric solution for 48 hours at 20°C by raising the water table slowly on a Haynes' apparatus containing the packed soil cores.

Once the soil had been saturated, as described above, the cores were placed on a mesh support over a saturated solution of CaCl_2_ in a desiccator. The desiccators were kept at a constant temperature of 20 ± 1°C, to give a relative humidity of 30%. The air in the desiccator was circulated with battery‐driven fans.

In total, there were six treatments: control (in distilled water, T_DW_), with root exudates at 1.25 g C kg^−1^ dry soil (T_1.25_), 2.5 g C kg^−1^ dry soil (T_2.5_) and 5.0 g C kg^−1^ dry soil (T_5.0_), sterilized in distilled water (T_DW + Hg_) and finally sterilized root exudates at 2.5 g C kg^−1^ dry soil (T_2.5 + Hg_). The evaporation loss in terms of volumetric water content in the core was measured regularly (hourly) until the water content was constant with time. The cores were removed from the desiccator briefly to measure their mass.

### *Soil water release characteristics*

The water release characteristics were measured on duplicate samples. Plastic cylindrical cores of 50 mm in diameter and 25 mm long were filled homogenously to two‐thirds height with air‐dry 2‐mm‐sieved loamy sand soil to a packing density of 1.5 g cm^−3^. The exudate treatments were applied to these cores as described above. The saturated soil in the cores was incubated at saturation for 14 days in the dark at room temperature (20 ± 1°C). After 14 days of incubation, all the cores were equilibrated at eight matric potentials varying from −1, −3, −10, −30, −100, −300, −500 and −1500 kPa. To equilibrate samples at higher matric potentials (−1 to −30 kPa) we used a ceramic suction plate for 5–9 days, whereas samples at lower matric potentials (−100 to −1500 kPa) were equilibrated in a pressure chamber (plate apparatus) for 14–36 days, or longer for lower matric potentials of −500 and–1500 kPa. Three replicates were measured for each of the six treatments at each matric potential. At equilibration, the wet soil weight was recorded and water contents were calculated following oven drying at 105°C for 48 hours.

### *Estimation of hydraulic diffusivity*

Hydraulic diffusivity is given by dividing hydraulic conductivity by the derivative of the water release curve; its advantage is that its range, or variation, is smaller than that of hydraulic conductivity (Hillel, 1980). We fitted a linearized solution of the desorption process proposed by Black *et al*. (1969) to our data:

(1) $$E_{i}=2\theta_{i}{\left[\frac{t\;D_{\mathrm{av}}}{\pi \;}\right]}^{1/2},$$

where *E*_*i*_ is the cumulative evaporation (cm) and *θ*_*i*_ is the water content at time *t*, *D*_av_ is the weighted mean diffusivity (Black *et al.,* 1969), which was assumed to be constant for a particular soil (Parlange *et al.,* 1992). This equation applies to a semi‐infinite column of soil, which is approximated in the early stage of drying when the water content at the base of the core is not yet reduced. We also assume that the water content at the surface had dried instantly to the final water content.

In practice, diffusivity is a function of soil water content, *D*(*θ*). The weighted mean diffusivity, *D*av, is related to *D*(*θ*) as follows:

(2) $$D_{\mathrm{av}}=\frac{1.85}{{\left(\theta_{i}-\theta_{s}\right)}^{1.85}}\int_{s}^{i}{\left(\theta_{i}-\theta \right)}^{0.85}\;D\left(\theta \right)d\theta ,$$

where *θ*_i_ is the initial water content and *θ*_s_ is the water content at the soil surface (Black *et al*., 1969; Parlange *et al*., 1992). The weighted mean diffusivity can be used to interpret time‐series data relating to soil water content (Parlange *et al*., 1992). Ritchie *et al*. (2009) used an averaged description of water transport to describe stage 2 evaporation (this is evaporation from unsaturated soil). They called this a ‘functional’ approach instead of a truly ‘mechanistic’ model where, for example, soil water diffusivity depends on water content. Such functional approaches based on constant soil water diffusivity have been used by Passioura (1991) to explore the effects of differences in macroscopic soil structure on water uptake by roots. For our study, an averaged diffusivity is sufficient to discuss the effects of microbial activity on evaporation. As in the case of Ritchie *et al*. (2009), we are considering stage 2 evaporation and our data are not affected by the movement of water under the influence of gravity.

## Statistical analysis

To compare the effect of the experimental treatments on the soil drying curves, we chose a four‐parameter logistic curve and the independent variable is the square root of time in hours; the equation is given by:

(3) $$\theta =A+\frac{C}{\left(1+e^{B\left(\sqrt{t}-M\right)}\right)},$$

where *A*, *B*, *C* and *M* are adjustable fitting parameters. We chose a logistic curve because the initial drying did not provide a linear relationship with $\sqrt{t}$. The logistic fit enabled us to identify this condition, which was assumed to occur near the point of inflection *M*. Parallel curve analysis consisted of a series of steps with the fitting of increasingly complex models, and finally choosing the relation that best explained the data. First, a single curve with one set of estimated parameters was fitted. The parameters can be split into linear (*A* and *C*) and non‐linear (*B* and *M*) sets and the next step fits separate intercepts and separate *A* parameters, representing a lower asymptote. In the next step, we fitted separate *C* parameters, which represent the available water being evaporated. In the final step we fitted both *B* and *M* as separate parameters to each treatment set of data, where *B* is the slope parameter that determines how fast the drying is and *M* is a location parameter that positions the curve on the $\sqrt{t}$ axis. The analysis was summarized in the accumulated analysis of variance table and at the end of the fitting process we also checked the residuals for normality and homogeneity of variances. The best model to fit to our data had separate parameters for each of the different treatments. The parameters that were of most interest were *B*, representing the slope, and *M*, which represents the mid‐point of the region where *θ* is linear with$\;\sqrt{t}$. Diffusivity was calculated for each of the separate replicate samples over a range of $\sqrt{t}$ where water loss was linear with $\sqrt{t}$. Linearity was confirmed by inspection, although the location of the linear region of the curve was indicated by *M*.

The water release data were fitted by the Van Genuchten Equation:

(4) $$\theta_{\psi }=\theta_{r}+\frac{\theta_{s}-\theta_{r}}{{\left[1+{\left(\alpha \psi \right)}^{n}\right]}^{m}},$$

where *θ*_ψ_ is the water content at matric potential ψ, *θ*_*r*_ is the residual water content, *θ*_*s*_ is the water content at saturation and *α*, *n* and *m* are fitting parameters. All of these parameters were estimated with curve fitting and we used the constraint of *m* = 1–1/*n* (van Genuchten *et al*., 1980). For each treatment, separate curves were required. These data were also analysed with analysis of variance.

We used Genstat (VSN Int. Ltd, Hemel Hempstead, UK, or Payne, 2015) to fit all the curves described above to our data and for all statistical analysis.

## Results

**Figure 1:**
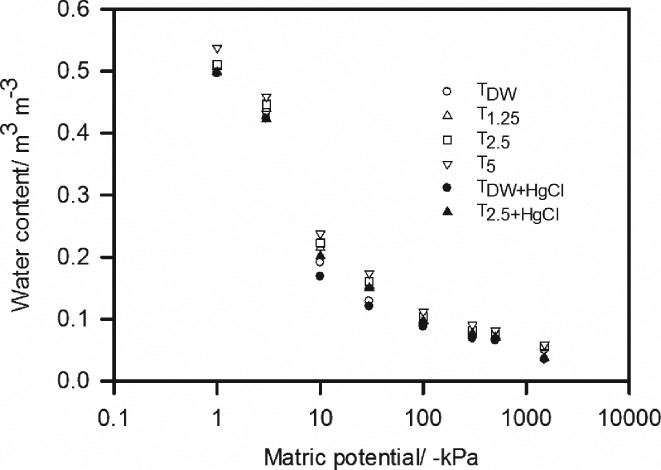
The water release characteristics of soil samples with different treatments. In the interests of clarity, the parameter values are given in Table 1. Analysis of variance of these data (Table 2) showed that the main effects of soil treatment and water potential, as well as their interaction, were all significant at P < 0.001.

The incubation of soil with artificial root exudates had a small, but significant, effect on the water release characteristics (Figure 1). The water release characteristics in Figure 1 were fitted to the van Genuchten equation (Equation (4)) and the parameter values are listed in Tables 1 and 2. Analysis of variance of these data showed that the main effects of soil treatment and water potential, as well as their interaction, were all significant at *P* = 0.001 (Table 2).

**Table 1:** Parameters of the van Genuchten equation (Equation (4)) for the water release curves plotted in Figure 1

Treatment	van Genuchten parameter				Percentage of variance accounted for
	*θs*	*θr*	*α*	*n*	
T_DW_	0.508 (0.0047)	0.0491 (0.00443)	1.481 (0.032)	3.373 (0.124)	99.8 (*P* < 0.001)
T_1.25_	0.509 (0.0055)	0.0413 (0.00704)	1.428 (0.039)	3.014 (0.135)	99.7 (*P* < 0.001)
T_2.5_	0.515 (0.0056)	0.0428 (0.00759)	1.407 (0.04)	2.988 (0.140)	99.7 (*P* < 0.001)
T_5.0_	0.541 (0.0057)	0.0334 (0.00942)	1.439 (0.042)	2.748 (0.125)	99.7 (*P* < 0.001)
T_DW + Hg_	0.499 (0.0077)	0.0490 (0.00634)	1.529 (0.005)	3.508 (0.202)	99.4 (*P* < 0.001)
T_2.5 + Hg_	0.502 (0.0074)	0.0271 (0.0111)	1.485 (0.011)	2.808 (0.171)	99.4 (*P* < 0.001)

The standard error of the coefficient is shown in brackets. The treatments are as follows: control (in distilled water, T_DW_), with root exudates at 1.25 g C kg^−1^ dry soil (T_1.25_), 2.5 g C kg^−1^ dry soil (T_2.5_) and 5.0 g C kg^−1^ dry soil (T_5.0_), sterilized in distilled water (T_DW + Hg_) and finally sterilized root exudates at 2.5 g C kg^−1^ dry soil (T_2.5 + Hg_).

**Table 2:** Results from the analysis of variance of the water release data shown in Figure 1

Source of variation	d.f.	Sum of squares	Mean square	*F*	*F* probability
Block stratum	2	2.38E‐06	1.19E‐06		0.41
Block • Sample stratum					
Treatment	5	1.91E‐02	3.81E‐03	1302.7	<0.001
Matric potential	7	3.94E+00	5.63E‐01	1.92E+05	<0.001
Treatment • Matric potential	35	6.45E‐03	1.84E‐04	62.94	0.001
Residual	94	2.75E‐04	2.93E‐06		
Total	143	3.97E+00			

Both the main effect and the interaction were significant at *P* < 0.001. d.f., degrees of freedom.

**Figure 2:**
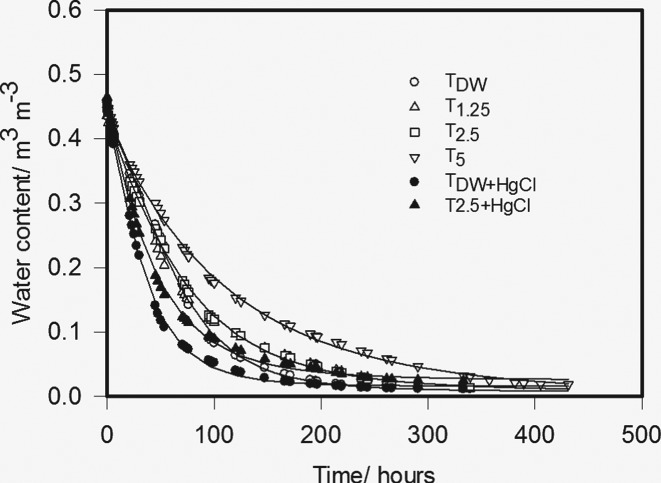
Water content plotted against time for the different experimental treatments. These data were fitted by Equation (3) and the fitted curves are shown. The fitted parameters are given in Table 3.

**Figure 3:**
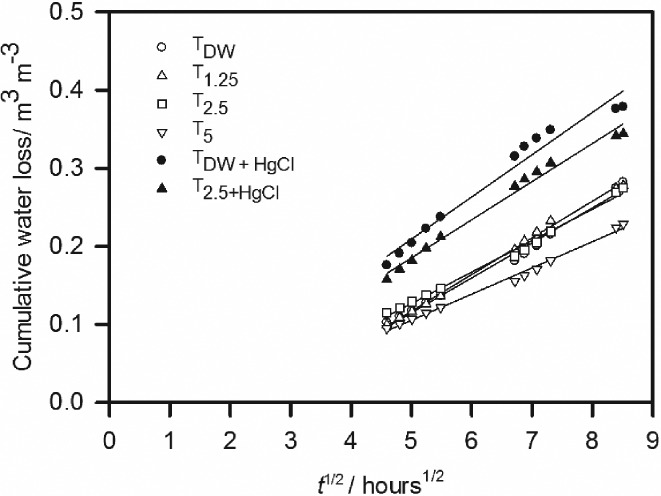
The relation between the cumulative water loss and $\sqrt{t}$. These data correspond to the region where the water loss is expected to be a linear function of $\sqrt{t}$. We used the value of M, in Equation (3), as an approximate guide to this region (i.e. between 5 and 7).

The effect of the experimental treatments on the soil drying curves obtained from the desiccator experiment was pronounced (Figure 2). The parameter values for Equation (3) are listed in Table 3 and the fit to our data accounted for 99.8% of the variance in the data. Different coefficients were required for the different treatments (*P* < 0.001). The logistic form of Equation (3) was required because early in the evaporation experiment water loss was not a linear function of $\sqrt{t}$. This was probably because the saturated calcium chloride solution in the desiccators did not absorb the excess humidity at a sufficient rate, which would have led to variable boundary conditions with, initially, RH > 30%. After long periods of time, the relation between water loss and $\sqrt{t}$ was no longer linear. This is likely to be because of soil drying at the base of the core and the failure in the semi‐infinite‐column condition. The value of *M* is the approximate mid‐point of the linear relation between *θ* and $\sqrt{t}$. The parameter *M* has values between approximately 5 and 7, and our assumption, that during those times when the relation between *θ* and $\sqrt{t}$ was linear, was confirmed by inspection (i.e. within the first 2 days for all treatments and within the first day for some treatments, T_DW + Hg_ and T_2.5 + Hg_). In Figure 3, we plotted the earliest cumulative drying data where there was a linear relation with $\sqrt{t}$, and show the calculated average diffusivity with Equation (1) in Table 4. Table 4 also gives the mean and range in water contents associated with each estimate of average diffusivity. The largest diffusivities are associated with the smaller water contents. Diffusivity was smaller in treatments with larger amounts of added synthetic exudates and largest in treatments with added mercuric chloride.

**Table 3:** Parameter values for Equation (3) when fitted to the drying curves for the different treatments, which are plotted in Figure 1

	Parameter values of Equation (3) (SE in brackets)			
Treatment	A*	*C*	*B*	*M*
T_DW_	0.00804 (0.00214)	0.4418 (0.00804)	−0.5003 (0.014)	7.19 (0.0588)
T_1.25_	0.01593 (0.00288)	0.4596 (0.00949)	−0.3776 (0.0135)	6.58 (0.103)
T_2.5_	0.00823 (0.0033)	0.4840 (0.0106)	−0.3516 (0.0128)	6.74 (0.108)
T_5.0_	0.00126 (0.0046)	0.5382 (0.0156)	−0.2481 (0.0101)	7.34 (0.175)
T_DW + Hg_	0.01592 (0.00175)	0.4731 (0.00811)	−0.5447 (0.0171)	4.94 (0.0798)
T_2.5 + Hg_	0.02585 (0.00219)	0.5089 (0.01188)	−0.4119 (0.0140)	5.01 (0.123)

Parallel curve fitting accounted for 99.8% of the variance and confirmed that the best fit to the data was obtained with different coefficients for each treatment. Accumulated analysis of variance, following grouped regression, showed that each treatment required separate parameters (*P* < 0.001). A*, lower asymptote; *C*, intercept represents available water being evaporated; *B*, slope parameter that determines how fast the drying is; *M*, location parameter represents the mid‐point. Treatment abbreviations are explained in the text and Table 1.

**Table 4:** Average diffusivities estimated with Equation (1) and the data in Figure 3. These are the mean values for the diffusivities calculated for each individual replicate

Treatment	Log_10_ (*D*/cm^2^ day^−1^)	Porosity	Back‐transformed diffusivity (cm^2^ day^−1^)	Water content /cm^3^100 cm^−3^		
				Min	Max	Mean
T_DW_	0.61	0.51	4.07	1.3	8.7	3.6
T_1.25_	0.67	0.51	4.68	2.1	11.9	6.0
T_2.5_	0.55	0.51	3.55	2.1	12.4	6.0
T_5.0_	0.32	0.54	2.08	5.7	18.0	10.7
T_DW + HgCl_	0.96	0.50	9.12	1.3	5.4	2.5
T_2.5 + HgCl_	0.79	0.50	6.17	2.7	9.2	5.0

The least significant difference in log_10_ *D* (LSD for *P* = 0.05) is 0.072. The final soil porosity data are also listed (LSD = 0.002 for *P* = 0.05). The minimum (Min), maximum (Max) and mean water contents corresponding to the range of water contents associated with estimated diffusivity are also given. Treatment abbreviations are explained in the text.

## Discussion

### *Soil porosity*

The effects of microbial activity on soil porosity in this research were small. Helliwell *et al*. (2014), who studied soil that was kept saturated for the duration of the experiment, reported much greater effects of microbial stimulation on soil porosity. They found that microbial stimulation, by the addition of glucose to a soil similar in texture to that used in this study, resulted in an increase in porosity from approximately 38 to 54% (estimated from X‐ray imaging). In contrast, the largest porosity we found was for T_5_, which has a porosity of 54% in comparison with 49% in the treatment designed to limit microbial activity (T_DW + Hg_). The change in porosity values we observed in our experiment was small and suggests that the effect of microbial activity on soil structure in this study was also small. The small range in porosity in this research compared with that of Helliwell *et al*. (2014) is probably because our soil samples were unsaturated, whereas Helliwell *et al*. (2014) investigated saturated soils. The expansion of trapped gaseous emissions in bubbles in saturated soils, such as those described by Helliwell *et al*. (2014), is likely to alter both porosity and structure. Bubbles are not likely to form in unsaturated soil.

### *Hydraulic properties*

In common with previous studies, we found that microbial activity impeded the transport of water through soil, as shown by the diffusivity data in Table 4. Treatments with the greatest addition of exudate have the lowest diffusivity and those treated to stop microbial activity have the highest diffusivity. Soil drying curves in Figure 2 also show that the rate of soil drying is much slower in the treatments designed to stimulate microbial activity and faster in those treatments designed to suppress microbial activity. The effects of the treatments on the water release characteristics are statistically significant (Figure 1 and Table 2), although they are small in comparison with those reported by Read *et al*. (2003) and Ahmed *et al*. (2014) for the effect of exudates, and by Or *et al*. (2007) for the effects of extracellular polymeric substances.

### *Implications for roots*

Gao *et al*. (2017) showed that additions of the same synthetic exudates used in this work to the same soil altered the microbial community in both structure and quantity. Our data show that the flow of water through soil is also impeded; Figure 2 and Table 4 show that diffusivity was halved by increased microbial stimulation, which has been widely reported (Or *et al*., 2007). We also show that the flow of water can be increased by suppressing microbial activity with the addition of mercuric chloride. Our estimated mean diffusivity of the control soil more than doubled from 4.1 to 9.1 cm^2^ day^−1^ with the addition of mercuric chloride. The key implication is that much of the reported hydraulic data in the literature is affected by the background microbial activity that is present during the measurement process.

**Figure 4:**
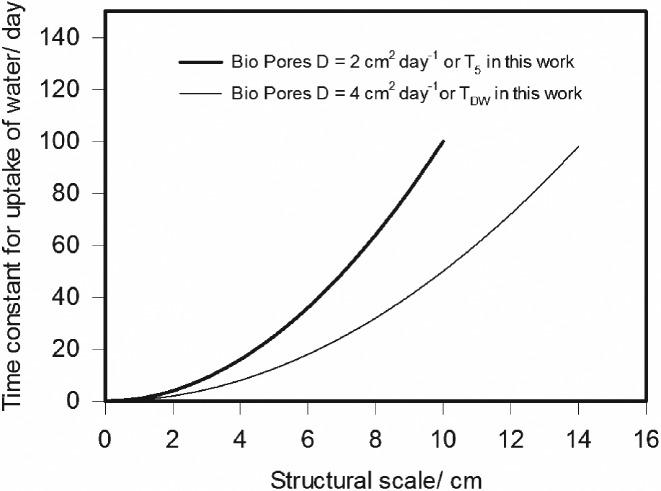
The time constant for water uptake (i.e. the time taken for roots to extract half of the available water) plotted against structural scale. These data represent the case for bio‐pores (Passioura, 1991). The difference between the two curves represents the range of values of the time constant for water uptake possibly related to the effects of exudates at 5 g kg^−1^ soil (2 cm^2^ day^−1^) compared with the control (4 cm^2^ day^−1^).

Passioura (1991), assuming a mean diffusivity of 2 cm^2^ day^−1^, concluded that it was macroscopic soil structure (e.g. aggregated or blocky) or the distribution of roots within soil that was most likely to limit water uptake by roots, and not the movement of water through bulk soil. In our study, the least conductive soil obtained by adding the largest amount of exudates (5.0 g C kg^−1^ dry soil, T_5.0_) had a diffusivity of 2.08 cm^2^ day^−1^, which was close to half of that estimated for the soil without any added exudates (4.07 cm^2^ day^−1^). It seems likely that any possible effect of exudates (with respect to water uptake by roots) on the soil we studied must be related to reducing the rate of water sorption from soil. In Figure 4 we have replotted the relation between the time constant for water uptake (i.e. the time taken for the roots to take up half of the available water) and the structural scale derived by Passioura (1991) for *D* = 2 cm^2^ day^−1^ (i.e. T_5_) alongside the same curve for *D* = 4 cm^2^ day^−1^ (i.e. T_DW_). The effect of root exudates was to double the time constant for water uptake by roots growing in bio‐pores. Furthermore, because the time constant for water uptake approximates to *w*^2^/4*nD* where *n* = 1, 2 or 3 for slabs, prisms and cubes and *w* is the structural scale (see Passioura, 1991), the effect of root exudates in halving *D* is equivalent to making prisms behave like slabs. This assumes that the effects of exudates are uniformly distributed in soil; however, the proportion of soil influenced by roots is restricted. Nevertheless, the effect of exudates in restricting water uptake might be an important and beneficial trait. Wheat‐breeding programmes have reduced xylem conductance as a strategy for improving water use efficiency in arid regions (Richards *et al*., 2010), allowing water use to be distributed more uniformly in the growing season. It is reasonable to expect that a doubling of the time constant for water uptake could play an important role in this endeavour.

In contrast to the more immediate effects of recently applied mucilage in increasing conductance (Ahmed *et al*., 2014), we found that microbial activity had the effect of reducing conductance. Our data are consistent with those of Colica *et al*. (2014), who showed that the hydraulic conductivity decreased with increasing additions of high‐molecular‐weight compounds. At high water potentials, such as those studied by Ahmed *et al*. (2014), root mucilage can increase the soil water content, and this effect can increase the hydraulic conductance of soil, thereby increasing root water uptake. Simulations show that root mucilage can help plants to sustain transpiration for up to 42 hours as the soil dries (Carminati *et al*., 2015). In our study, we measured soil drying over a similar period, although our treatments included the growth of microbial communities (Gao *et al*., 2017) or their suppression (Wolf *et al*., 2013). It seems that the effects of root‐exuded mucilage can be split into short‐term effects from the physical effects of surface tension and viscosity (Kroener *et al*., 2014), and the longer‐term effects that arise because exudates stimulate microbial activity. Our data are most relevant to the long‐term effects that arise because microbial activity blocks soil pores (e.g. Wolf *et al*., 2013) or modifies wettability (e.g. Hallett & Young, 1999). The longer‐term effects of microbial activity are more commonly associated with the mineralization of nutrients, particularly in the production of nitrate. However, as we have discussed above, the reduction in diffusivity may well play an important role in moderating the use of soil water reserves in arid conditions, or in climates with well‐defined wet and dry seasons. Tardieu (2012) has observed that almost any trait may conserve water in the right circumstances.

## Conclusions

The addition of synthetic root exudates to a sandy soil reduced the hydraulic diffusivity. Compared with a control soil with no added exudates, the addition of 5 g C kg^−1^ of soil halved the diffusivity from 4 to 2 cm^2^ day^−1^. Suppression of microbial activity with the addition of mercuric chloride to soil increased diffusivity more than twofold in comparison with the control, from 4 to 9 cm^2^ day^−1^. Analysis of root water uptake suggests the effect of the decrease in diffusivity is comparable to a shift in the soil structural unit, for example moving from a prismatic soil to slab structure.
